# Gynaecological cancers and leptin: A focus on the endometrium and ovary

**Published:** 2018-03

**Authors:** A Ray, J Fornsaglio, S Dogan, S Hedau, D Naik, A De

**Affiliations:** Lake Erie College of Osteopathic Medicine, Seton Hill University, Greensburg, PA 15601, United States; School of Natural & Health Sciences, Seton Hill University, Greensburg, PA 15601, United States; Department of Medical Biology, School of Medicine, Yeditepe University, Istanbul, 34755, Turkey; National Institute of Cancer Prevention & Research (ICMR), Noida 201301, U.P., India; National Institute of Traditional Medicine (ICMR), Belagavi 590010, Karnataka, India; Ovarian Cancer Laboratory, Veterans Affairs Medical Center, Kansas City, MO 64128, United States

**Keywords:** Leptin, obesity, ovarian epithelium, pelvic mass, uterine cancer

## Abstract

Obesity has an influence on the risk and prognosis of different types of cancers of the female reproductive tract. In the uterus, a common site for neoplasms is the endometrium, the inner lining tissue. Generally, obesity has been documented to be involved in endometrioid carcinoma of the endometrium. Obesity may influence the cancer risk by various mechanisms such as chronic inflammation, dysregulation of sex hormones and abnormal secretion of hormone-like cytokines or adipokines from adipose tissue. One of the important pro-inflammatory adipokines is leptin, which acts via its transmembrane receptors (Ob-R). In normal conditions, leptin functions in the hypothalamic anorexigenic pathway to maintain the energy homeostasis. Conversely, in obesity, leptin participates in the pro-inflammatory processes. Several clinical studies have suggested that leptin and Ob-R play a role in the pathological processes of endometrial cancer. In different endometrial cancer cell lines, laboratory findings also have demonstrated leptin’s link to various neoplastic phenomena such as cellular proliferation, angiogenesis, and oestrogenic activity. Furthermore, endometrial cancer risk could be increased in ovarian pathology like polycystic ovary syndrome, which is commonly associated with obesity. It is noteworthy that leptin participates in both physiological and pathological conditions of the ovary. Leptin has shown pro-tumorigenic effects in both in-vitro and in-vivo studies. Generally, reduced serum leptin levels have been observed in ovarian cancer patients. However, overexpression of leptin and Ob-R in ovarian cancer tissue has indicated aggressive disease. Understanding the role of leptin-related intracellular signalling pathways in tumour development could be helpful in early cancer detection.

## Introduction

Gynaecological cancers include commonly occurring malignancies such as carcinomas of the uterine cervix (cervical cancer), endometrium and ovary as well as relatively rare malignancies of the fallopian tube, vagina, vulva, and choriocarcinomas. These cancers were responsible for more than 16% of all cancers and nearly 14% of all cancer deaths worldwide among women in 2012 (Constantinou and Tischkowitz, 2017). Overall, cervical cancer is the most common gynaecological cancer, followed by endometrial and ovarian cancers. However, in the Western world, endometrial cancer is the most common gynaecological malignancy.

It has been found that endometrial cancer risk was directly correlated with obesity (Garmendia et al., 2013; Kendall et al., 2015; Wise et al., 2016; Shaw et al., 2016). In addition, early-life obesity was associated with a moderately increased risk of endometrial cancer later in life. On the other hand, a recent report, which evaluated 36,794 postmenopausal women, has documented that endometrial cancer risk increased in weight gain, whereas weight loss was associated with a lower risk (Luo et al., 2017). Interestingly, in a number of studies, endometrial cancer patients exhibited higher blood levels of triglycerides compared to controls (Lindemann et al., 2009; Seth et al., 2012; Sun et al., 2016). Furthermore, it is known that obesity is a poor prognostic factor for endometrial and other cancer patients alike. Obesity has been shown to be associated with increased mortality among women with endometrial cancer (Shaw et al., 2016; Secord et al., 2016).

Like endometrial cancer, several investigators reported that overweight/obesity was associated with an increased risk of ovarian cancer and poor prognosis (Poorolajal et al., 2014; Liu et al., 2015; Craig et al., 2016). It is worth mentioning that depending on the cellular origin and histological characteristics, ovarian cancers are classified into various subtypes. The majority of the primary tumours arise from the surface epithelium; the three main histological types are serous (most common), mucinous and endometrioid tumours. Obesity may increase the risk of only certain histological subtypes. After analyzing 13,548 cases, the investigators concluded that higher bodyweight was associated with increased risk of borderline serous, low-grade invasive serous, invasive endometrioid, and invasive mucinous tumours (Olsen et al., 2013). Similarly, the findings of another study suggested a positive correlation between higher bodyweight and the risk of low-grade serous ovarian cancers (Dixon et al., 2016).

Overweight or obese patients with cervical cancer also showed poor prognosis (Clark et al., 2016; Choi et al., 2017). In general, the most common histological subtype of cervical cancer is squamous cell carcinoma, which constitutes approximately 80% of cases; and the next common subtype is adenocarcinoma. Two early case-control studies revealed that obesity increased the risk of adenocarcinoma (Brinton et al., 1987; Lacey et al., 2003). Other subsequent studies also documented an association between overweight/obesity and an increased risk of cervical cancer (Ulmer et al., 2012; Lee et al., 2013; López-Hernández, 2013; Webb, 2013; Poorolajal and Jenabi, 2016). Likewise, obesity has been shown to increase the risk of developing vulvar cancer, as demonstrated in two recent studies that analyzed a large number of subjects. After an average 14 years follow-up, 898 vulvar cancer cases were registered in a cohort of 1.3 million women aged 49-65 years. Obesity was found to be a significant risk factor (Coffey et al., 2016). In another study where 201,469 women were followed for an average 13.8 years, there were 370 cases of vulvar neoplasms including 170 invasive tumours. In this study, obesity increased invasive cancer risk (Brinton et al., 2017).

Among all gynaecological malignancies, obesity has been associated most frequently with endometrial cancer, particularly type-I or endometrioid carcinoma of the endometrium, which is the most common and usually well-differentiated endometrial cancer. An excess adipose tissue in obesity may influence the risk of cancer development by a number of mechanisms, e.g., chronic inflammation, dysregulation of sex hormones, insulin resistance, altered immune response, and abnormal secretion of cytokines (Iskander et al., 2013). Dysfunctional adipose tissue releases an abnormal amount of pro- inflammatory cytokines such as interleukin-6 (IL-6) and tumour necrosis factor-α (TNF-α), along with other proteins like leptin and plasminogen activator inhibitor-1 (PAI-1), which may contribute to an alteration of crucial signalling pathways (Divella et al., 2016). Nevertheless, this inflammatory situation leads to a state of insulin resistance and anomalous responses of both innate and adaptive immunity, and ultimately a tumorigenic environment (Conroy et al., 2016).

Leptin is a 16 kDa protein and an important hormone-like cytokine or adipokine. It is mainly secreted from adipose tissue and primarily involved in the maintenance of energy homeostasis by influencing the central anorexigenic pathway (Ray, 2012). However, hormonal functions of leptin are not only restricted to the central hypothalamic area to control appetite. This adipokine has a number of effects in the peripheral tissues (Dogan et al., 2010). In obesity, it participates in the pro-inflammatory processes and perpetuates the state of insulin resistance. Leptin acts via transmembrane receptors (Ob-R), which are present in at least 6 alternatively spliced isoforms (Ob-Ra-f). The long form Ob-Rb appears to be important for leptin’s role in weight regulation and pro-inflammatory effects. Here, we describe the role of leptin in gynaecological cancers. To achieve this, a systematic literature search was carried out primarily in PubMed.

## Effect of leptin in endometrial cancer

It is known that overweight/obesity is a major risk factor for the development of endometrial cancer and the mortality associated with it. Leptin is one of the important adipokines, which probably plays a key role in this pathological process (Uchikova et al., 2015). In recent times, a number of pathological and molecular differences have been revealed between type-I and type-II endometrial cancers. For instance, type-I tumours usually originate in an environment of endometrial hyperplasia, whereas type-II tumours are typically linked with pathognomonic features such as endometrial atrophy, high grade, and common occurrence of p53 mutations. Both type-I tumors and precursor lesion hyperplasia are commonly observed in obesity. Nevertheless, it has been hypothesized that leptin signalling and its crosstalk may also be associated with the more aggressive and poor prognostic type-II endometrial cancer (Daley-Brown et al., 2015).

### Findings from in vitro studies

In an in vitro study, leptin treatment resulted in increased proliferation of hormone-responsive Ishikawa/ECC1 type-I endometrial cancer cells (Sharma et al., 2006). Moreover, the investigators found that leptin potently induced invasion of endometrial cancer cells in a matrigel invasion assay. In another study, Ishikawa cells were treated by leptin at various concentrations at different time points (Gong et al., 2007). The study observed that leptin stimulated the proliferation of Ishikawa cells. In addition, the experiments documented that extracellular signal-regulated kinase 1/2 (ERK1/2) phosphorylation was enhanced significantly in Ishikawa cells after treatment of 100 ng/ml leptin. Like the abovementioned study (Gong et al., 2007), Ishikawa endometrial cancer cells were treated by leptin with various concentrations for different durations (Liu et al., 2011). The results showed that leptin induced the phosphorylation of signal transducer and activator of transcription 3 (STAT3) and the activation of ERK1/2 in a time- and dose- dependent manner. In addition, leptin was found to potently induce the invasion of cancer cells in a matrigel invasion assay. Remarkably, leptin treatment stimulated the proliferation and invasion of SPEC-2 cells (type-II endometrial cancer cell line) (Wu et al., 2012).

In another study comparing cancer cell lines – low-grade type-I Ishikawa cells, high-grade type-I SK-UT2 cells, and type-II AN3CA cells, with benign endometrial epithelial cells, all endometrial cancer cell lines expressed higher levels of Ob-R in contrast to benign cells (Carino et al., 2008). Furthermore, leptin in a dose-dependent manner regulated vascular endothelial growth factor (VEGF), IL-1β, leukemia inhibitory factor (LIF) and their respective receptors, VEGFR2, IL-1R type I (IL-1R tI) and LIFR (Carino et al., 2008). Leptin’s effects on proangiogenic molecules also were more evident in malignant cells compared to benign cells. Leptin induced a greater increase in VEGF/VEGFR2 and LIF levels in cancer than in benign cells. The investigators reported that mammalian target of rapamycin (mTOR, mainly linked to mitogen-activated protein kinase/MAPK) played a central role in leptin regulation of these cytokines and their receptors.

In a study where leptin was added to various human endometrial cancer cell lines, e.g., Ishikawa cells, HEC-1A cells (moderately differentiated adenocarcinoma), RL95-2 cells (moderately differentiated adenosquamous carcinoma), and AN3CA cells, leptin stimulation resulted in increased expression of cyclooxygenase (COX)-2 mRNA and prostaglandin E2 (PGE2) production (Gao et al., 2009). The role of COX-2 in prostaglandin biosynthesis via arachidonic acid metabolism in inflammation is noteworthy. The investigators also showed that leptin stimulated cell proliferation and induced activation of STAT3, ERK1/2, and AKT dose-dependently. On the other hand, in a co-culture model of endometrial fibroblasts and Ishikawa cells, aromatase mRNA expression was increased in treatment with 100 ng/ml leptin (Liu et al., 2013). Besides, estradiol synthesis was induced when precursor hormone androstenedione was added to the culture medium treated with leptin. In another study, leptin treatment was associated with an up-regulation of cyclin D1, an important regulator for cell cycle progression, in Ishikawa cells (Catalano et al., 2009). A study, which investigated the role of leptin in the process of programmed cell death in endometrial carcinoma, observed that leptin induced a decrease in apoptosis in Ishikawa and HEC-1A cells (Zhou et al., 2015). Therefore, it is comprehensible that the presence of leptin in tumour microenvironment could play a substantial role by influencing a number of biological mechanisms such as inflammation, cellular proliferation, and evasion of apoptosis.

### Clinical evidence relating to leptin

All of the splice variants of Ob-R were shown to be expressed in the human endometrium (Kitawaki et al., 2000). In a study, both endometrial tissue and blood samples were collected from standard-weight, overweight and obese women with normal endometrial histology, along with samples from obese type-I endometrial cancer patients (Villavicencio et al., 2010). In endometrial samples with normal histology, epithelial cell proliferation was higher in the overweight and obese groups, and proliferation was positively correlated with serum levels of leptin. A significant increase in endometrial proliferation was found in cancer patients.

In a study where immunohistochemical expressions of leptin, Ob-R and hypoxia-inducible factor-1α (HIF-1α) were analyzed in endometrial cancer tissue, immunoreactivity for leptin and Ob-R protein was observed in 56.7% and 30.0% of endometrial cancer cases, respectively (Koda et al., 2007). Moreover, a positive correlation between leptin and Ob-R expression was noted, and the expression of HIF-1α showed a significant positive correlation with leptin and Ob-R. It is worth mentioning that HIF-1α has been demonstrated to be involved in tumour progression and poor prognosis (Masoud and Li, 2015). In a study on endometrial cancer specimens, the expression of leptin and Ob-R was associated with oestrogen receptor (ER) expression, lymph node metastasis, and poorer prognosis (Zhang et al., 2014).

A number of studies showed that high serum leptin level was associated with increased risk of endometrial cancer (Petridou et al., 2002; Cymbaluk et al., 2008; Wang et al., 2014; Cymbaluk-Płoska et al., 2018). Interestingly, Zhou et al. (2015) found that serum leptin concentration and cancer tissue expression of Ob-R correlated with degree of differentiation of endometrial carcinoma. For instance, expression levels of Ob-R were higher in poorly and moderately differentiated compared to well-differentiated endometrial carcinoma tissue samples. On the other hand, many investigators studied the circulating levels of leptin with another adipocyte-released cytokine, adiponectin. It is thought that adiponectin opposes the effects of leptin in obesity. Studies recoded that the risk of endometrial cancer was associated with increased leptin or decreased adiponectin levels (Ma et al., 2013; Luhn et al., 2013).

Overall, the uterus is an oestrogen sensitive organ. As mentioned earlier that leptin acts like a pleiotropic hormone and it has a number of effects in different tissue types, including hormone sensitive organs and endocrine glands. A recent report observed that Ob-R expression fluctuated in correlation with ER and progesterone receptor (PR) during different endometrial phases and pathological conditions, suggesting an oestrogen-dependent state of Ob-R in the endometrium (Méndez-López et al., 2017). An appropriate knowledge of interactions between oestrogen and leptin signalling pathways is helpful to understand the endometrial pathologies comprehensively.

## Ovarian cancers

**Figure 1:**
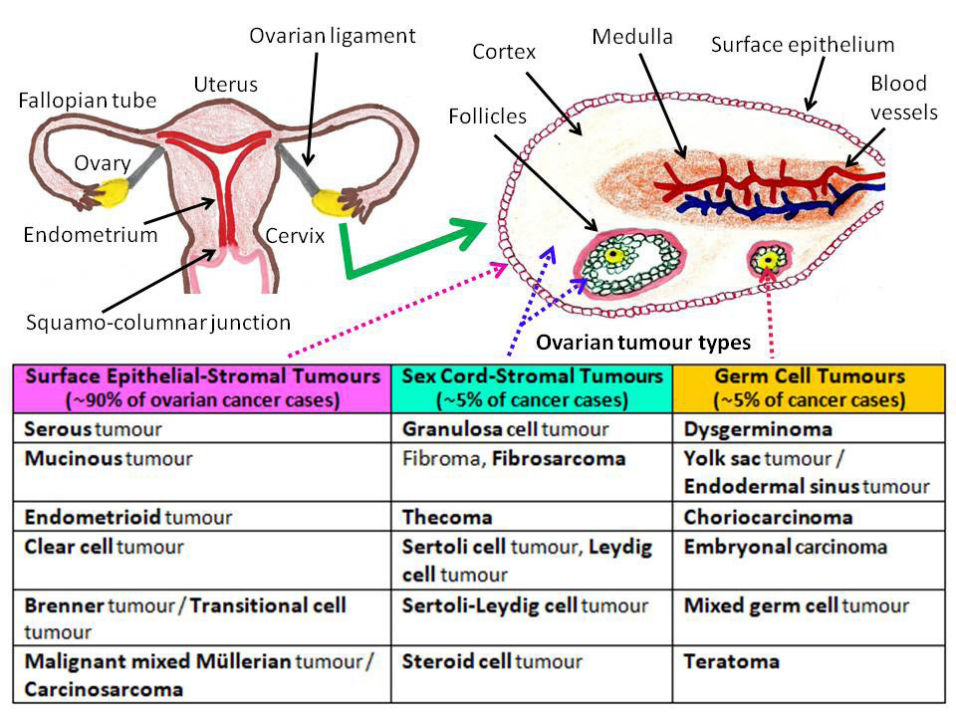
— Schematic representation of the female genital tract including ovarian histological diagram and relevant neoplastic characteristics.

Ovarian cancer is the seventh leading cancer and eighth cause of cancer mortality among women worldwide (Coburn et al., 2017). In general, tumours derive from one of the following three components of the ovary: surface epithelium, germ cells, and stromal cells (Figure 1). Surface epithelial tumours are further subdivided into benign, borderline, and malignant neoplasms. Moreover, the majority of ovarian cancers originate from the surface epithelial cells, and the most common histological type is serous carcinoma. On the basis of histopathology and molecular genetics, surface epithelial tumours are classified into five morphologically diverse types: high-grade serous, endometrioid, clear cell, mucinous, and low-grade serous carcinomas (Prat, 2017). Furthermore, it is now thought that the fallopian tube epithelium is the source of ovarian serous carcinogenesis (George et al., 2016; Labidi-Galy et al., 2017).

Interestingly, there are certain histological similarities among endometrial and ovarian carcinomas (Table I) (Koshiyama et al., 2014; Ellenson and Pirog, 2015). Among synchronous tumours of the female genital tract, the coexistence of primary cancers of the endometrium and ovary occurs frequently (Makris et al., 2017). Histologically, endometrioid carcinomas of the endometrium and ovary have been detected in the majority of cases (Soliman et al., 2004; Natee et al., 2006; Sozen et al., 2015). In addition, studies reported that patients with synchronous primary endometrial and ovarian cancers were generally premenopausal, nulliparous, and obese (Soliman et al., 2004; Jain et al., 2017). It is believed that risk factors for the development of these simultaneous cancers are hyperestrogenic conditions, and of these, obesity is one of the common hyperestrogenic conditions (de la Noval, 2016).

**Table I:** Broad classification of neoplasms of the endometrium and ovary.

	Endometrial carcinomas	Ovarian carcinomas
Type I cancer	Endometrioid carcinoma	Endometrioid carcinoma Clear cell carcinoma Mucinous carcinoma Low-grade serous carcinoma Transitional cell carcinoma*
Type II cancer	Serous carcinoma Clear cell carcinoma* Malignant mixed Müllerian tumour*	High-grade serous carcinoma Undifferentiated carcinomas Malignant mixed Müllerian tumour*

*Less common histological subtypes

### Leptin in physiological condition of the ovary

**Figure 2:**
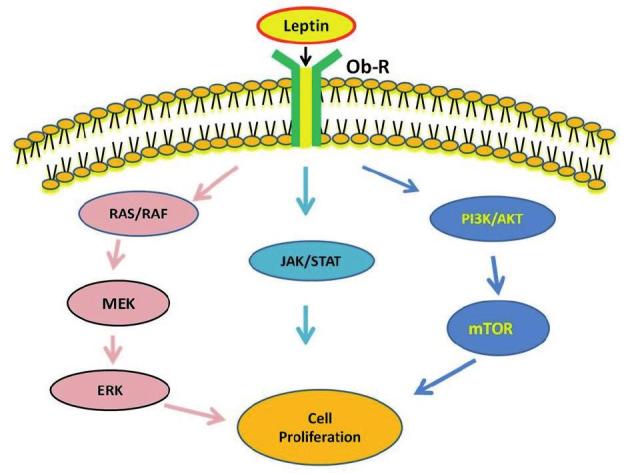
— Principal intracellular signalling pathways of leptin in connection with cellular proliferation. AKT: Protein kinase B/serine-threonine kinase, ERK: Extra-cellular signal-regulated kinase, JAK: Janus kinases, MAPK: Mitogen-activated protein kinase, MEK: Mitogen-activated protein kinase kinase, mTOR: Mechanistic/mammalian target of rapamycin, Ob-R: Leptin receptor, PI3K: Phosphatidylinositol-3-kinase, STAT: Signal transducer and activator of transcription

There are direct nutritional effects on both the ovaries and follicles (Dupont et al., 2012). Therefore, it is expected that leptin, as a part of the energy sensing mechanisms, may have an impact on the ovarian development. In fact, circulating or locally produced leptin possibly is associated with the direct modulation of ovarian function (Pérez-Pérez et al., 2015). Intracellularly, leptin signalling mainly involves activation of Janus kinase (JAK)/STAT, MAPK/ERK and phosphatidylinositol-3-kinase (PI3K) pathways (Pérez-Pérez et al., 2015) (Figure 2). In a study on Sprague–Dawley rats, daily administration of a low dose of leptin (3 μg) induced the ovulatory process and showed increased phosphorylation of both STAT3 and ERK1/2 (Di Yorio et al., 2013). In another study, granulosa cells from goose ovarian preovulatory follicles were cultured with leptin (Wen et al., 2015). The results suggested that leptin exerted its proliferative and anti-apoptotic effects on granulosa cells through the PI3K/AKT/mTOR signalling pathway. It is noteworthy that in the ovary, interactions between germ cells (oocytes) and surrounding granulosa cells are crucial for oocyte maturation (Dupont et al., 2012). Nonetheless, in line with the abovementioned findings (Di Yorio et al., 2013; Wen et al., 2015), Bilbao et al. (2015) reported that the daily administration of leptin induced follicular growth and ovulation in Sprague–Dawley rats. In the same way, Dupuis et al. (2013) revealed that the expression of Ob-R was markedly induced in the granulosa cells of ovulating follicles in C57BL/6N mice. On the other hand, leptin has been shown to stimulate ovarian estradiol release (Sirotkin et al., 2016; Ding et al., 2017). Overall, emerging evidence has demonstrated that leptin is involved in the control of reproductive functions by acting both directly on the ovaries and indirectly on the central nervous system (Catteau et al., 2016).

### Results of in vitro and in vivo studies

A number of studies observed that leptin treatment elicited significant cell growth/proliferation in different ovarian cancer cell lines, e.g., OVCAR-3, SKOV3, A2780, OV-90, and BG-1 cells (Choi et al., 2005; Nkhata et al., 2007; Chen et al., 2013; Ptak et al., 2013[a]; Xu et al., 2013).

Furthermore, in response to leptin, studies detected the overexpression of proteins such as cell cycle regulator cyclin D1, DNA replication associated proliferating cell nuclear antigen (PCNA), and anti-apoptotic Mcl-1, which are connected with the cellular proliferation (Nkhata et al., 2007; Ptak et al., 2013[a]; Chen et al., 2013). Regarding the anti-apoptotic effects in ovarian cancer cells, leptin has been documented to inhibit several components of apoptotic machinery like tumour necrosis factor receptor 1 (TNFR1), Bad, caspase-6, and caspase-3 (Ptak et al., 2013[a]; Ptak et al., 2013[b]). Primarily using ovarian endometrioid adenocarcinoma MDAH2774 cell line, Uddin and colleagues (2009) found that PI3K/AKT pathways were involved in the regulation of cell proliferation and prevention of apoptosis. Similarly, Chen et al. (2013) showed that the activation of PI3K/AKT and mitogen-activated protein kinase kinase (MEK)/ERK1/2 signalling pathways were implicated in the growth-stimulating effect of leptin in OVCAR-3 cells. In oestrogen-responsive BG-1 ovarian cancer cells, Choi et al. (2011) demonstrated that leptin induced cell proliferation as well as increased phosphorylation of STAT3 and AKT in a time- and dose-dependent manner. However, in their study, downregulation of ERα using small interfering RNA completely reversed leptin-induced growth of BG-1 cells. In addition, treatment with leptin resulted in a substantial increase in the cell growth of ERα-transfected OVCAR-3 and A2780 cells, whereas no significant difference was noticed among ERβ-transfected cells.

In OVCAR-3 cells, Hoffmann et al. (2016) found that leptin stimulated the migration of cells, which was mediated via the expression and activity of matrix metalloproteinase MMP-9. The results of a recent study have shown that leptin promoted MMP-7 expression in SKOV3 and OVCAR-3 cells, and MMP-7 gene silencing attenuated leptin-induced MMP-9 activation in SKOV3 cell line (Ghasemi et al., 2018). It is worth mentioning that MMPs can degrade/remodel the extracellular matrix (ECM) components during invasion and metastasis of cancer cells. Moreover, leptin has been shown to induce the expression of various MMPs, such as, MMP-2, MMP-9, and membrane type 1 matrix metalloproteinase (MT1-MMP/MMP-14) in human cancer cells (Barreto et al., 2015). Urokinase plasminogen activator (uPA) is another important serine protease that can degrade the ECM components and contribute to tumour cell migration (Barreto et al., 2015). Using different ovarian cancer cell lines (OVCAR-3, SKOV3 and CaoV-3), a recent report found that leptin induced ovarian cancer cell invasion via up-regulation of uPA (Ghasemi et al., 2017). In addition, their findings demonstrated the involvement of RhoA/ROCK (cytoskeletal regulator), PI3K/AKT, JAK/STAT pathways and nuclear factor kappa-B (NF-κB) activation in this process. In the same way, another study documented increased cancer cell migration/invasion through leptin-mediated activation of RhoA/ROCK, PI3/AKT and JAK/STAT3 pathways (Kato et al., 2015). In this study, the investigators used the p53-wild type, BRA-mutated HEY cells and the p53-null SKOV3 cells in order to simulate the two major molecular varieties – type I and II serous ovarian cancers. Furthermore, these investigators recorded that leptin contributed to the maintenance of stemness (i.e., stem cell characteristics) and the mesenchymal phenotype in ovarian cancer cells. In general, acquiring mesenchymal characteristics favors tumour progression and leptin possibly influences this crucial phenomenon (Ray and Cleary, 2017).

In an in vivo study, mouse ovarian cancer ID-8 cells were injected into the peritoneal cavity of the female C57B6 mice on a high energy diet (HED, 60 kcal% fat - fed ad libitum), along with control mice on a regular diet (RD, 7.2 kcal% fat - fed ad libitum) and a caloric restriction diet (CRD, 30% reduced from normal intake) (Al-Wahab et al., 2014). The HED group displayed the most extensive tumour formation at all the peritoneum-related organ and metastatic sites, accompanied with increased plasma levels of leptin and other inflammatory cytokines/hormonal substances, e.g., IL-6, monocyte chemoattractant protein-1 (MCP-1), VEGF, and insulin-like growth factor-1 (IGF-1). In contrast, the CRD group exhibited the reverse profile. The investigators suggested that the main tumour regressive effects of CRD might be associated with the decreased production of substances like IGF-1 and leptin (Al-Wahab et al., 2014). Subsequently, in a similar study, the investigators observed that the anti-diabetic agent metformin treatment in RD and HED mice resulted in a significant reduction in tumour burden as well as decreased levels of growth factors/cytokines such as IGF-1, IL-6 and leptin in both plasma and ascitic fluid, akin to the CRD mice (Al-Wahab et al., 2015). On the other hand, in another study, BG-1 cells were injected into the peritoneal cavity of female athymic nu/nu mice (Kasiappan et al., 2014). In this study, high-fat diet (HFD) stimulated BG-1 tumour growth by up to 6 folds. In addition, the data of this study suggested that HFD increased leptin to stimulate ovarian tumour growth in vivo.

### Clinical scenario: leptin’s influence

There are certain reports that suggest a positive relationship between polycystic ovary syndrome (PCOS) and ovarian cancer risk; though a link between PCOS and endometrial cancer has often been described. Nonetheless, studies also documented an increased risk of other cancers and specific histological types in subjects with PCOS (Table II). PCOS is the most common endocrine disease among women of reproductive age, affecting up to 10% of women worldwide. Perhaps, abnormalities in androgen biosynthesis-related enzymes and obesity (or related problems) are the fundamental disorders that connect other pathological characteristics such as excessive androgen production, insulin resistance, and altered adipose tissue metabolism. Since obesity is a common feature in PCOS, it is expected that women with PCOS may exhibit an impaired status of different adipokines including leptin. Overall, there are conflicting reports on the role of leptin in PCOS (Lecke et al., 2011; Jeon et al., 2013; Garruti et al., 2014; Cassar et al., 2015). In a recently published meta-analysis, the authors examined 991 women with PCOS and 898 controls (Zheng et al., 2017). They found elevated leptin levels in women with PCOS compared with non-PCOS controls. Similar results were also documented in other reports (Pehlivanov and Mitkov, 2009; Chang et al., 2011). Unlike the situation in endometrial carcinoma, the circulating levels of leptin in patients with ovarian malignancies generally have been shown to be lower in comparison with healthy or non-cancer controls (Sen et al., 2011; Jin et al., 2016; Horala et al., 2017). Frequently, the patients with ovarian cancer are diagnosed with advanced-stage disease.

**Table II:** Selected reports on polycystic ovary syndrome (PCOS)-associated cancer risk.

**Investigators, Cancer site/type and Study design**	**Subjects**	**Findings (in brief)**
Chumas et al. (1983) Endometrial cancer | Case report	19-year-old female	Development of malignant mixed Müllerian tumour of the uterus
Fearnley et al. (2010) National population-based case-control study (Australia)	Women under 50: 156 cases and 398 controls	Women with PCOS had a 4-fold increased risk of endometrial cancer
Gottschau et al. (2015) National Registry (Denmark, 1977-2012)	12,070 PCOS patients and cancer was diagnosed in 279 women with PCOS	4-fold increased risk for endometrial cancer, mainly type 1; also increased risk was found for kidney, colon and brain cancers
Iatrakis et al. (2006) Endometrial cancer (Greece, 1992-2004)	Women under 50: 81 patients with endometrial cancer and 100 controls	PCOS and diabetes were related to endometrial cancer
Kilicdag et al. (2011) Endometrial cancer (Turkey)	417 premenopausal women	PCOS and the presence of 2 or more polyps were associated with significant pre-malignant or malignant changes
Kim et al. (2016) Breast cancer | Population-based case-control study (United States)	1,508 women with breast, and 1,556 controls	Positive association between PCOS and premenopausal breast cancer
Maggio et al. (2007) Ovarian tumour | Case report	12-year-old girl with Turner syndrome	Developed PCOS in an ovary and a contralateral gonado-blastoma
Niu et al. (2016) Endometrial cancer | Case report	26-year-old woman with PCOS	Concurrent endometrial adenocarcinoma and clear cell carcinoma
Olsen et al. (2008) Ovarian cancer | Population-based case-control study (Australia)	1,276 cases with invasive epithelial ovarian cancer, 315 borderline malignant tumour and 1,508 controls	Serous borderline tumours were positively associated with a history of PCOS
Park et al. (2011) Endometrial cancer | Prospective study (Korea)	117 women with PCOS	Endometrial hyperplasia in 25 women (21.4%) [complex hyperplasia with atypia in 4 (3.4%)], and endometrial cancer in 2 women (1.7%)
Powolny et al. (1999) Ovarian cancer | Case repot	25-year-old woman	Carcinoma of the ovary coexisted with PCOS.
Prakansamut et al. (2014) Endometrial cancer | Cross-sectional study	52 women with PCOS and abnormal menstrual pattern	9 (17.3%) and 1 (1.9%) had endometrial hyperplasia and endometrial cancer, respectively
Press and Scully (1985) Endometrial cancer | Case report	27-year-old woman	Development of an endometrial malignant Müllerian mixed tumour
Schildkraut et al. (1996) Ovarian cancer | Population-based case-control study (United States)	476 subjects with epithelial ovarian cancer and 4081 controls	Ovarian cancer risk was found to increase 2.5-fold among women with PCOS
Shen et al. (2015) National population-based retrospective cohort study (Taiwan, 2000-2004)	3,566 PCOS patients and 14,264 controls	PCOS might increase the risk of endometrial cancer
Spremović-Radjenović et al. (1997) Ovarian cancer | Case report	16-year-old girl	PCOS with Sertoli-Leydig ovarian tumour
Wild et al. (2000) Long-term follow-up study (United Kingdom, 1930-1979)	A cohort of 786 women with PCOS was traced	Women with PCOS were at increased risk of endometrial cancer

Dissemination of cancer cells within the peritoneal cavity is common due to unique anatomical position of the ovary. It is thought that cancer progression, continued inflammation, and catabolic processes lead to decrease of serum leptin concentration (Grabowski et al., 2014). More precisely it can be suggested that the prolonged inflammatory response associated to the advanced stages of neoplastic disease is responsible for the energy metabolism impairment, thus down-regulating and exhausting leptin production (Macciò et al., 2009).

**Figure 3:**
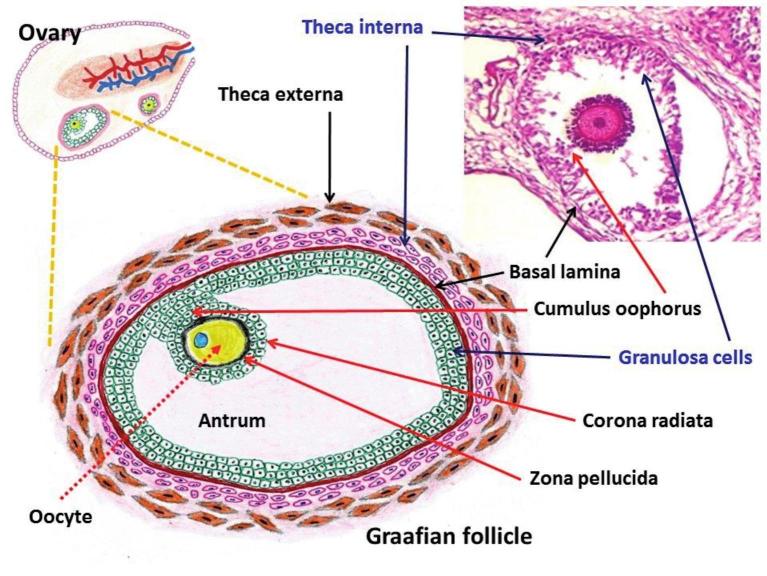
— Structure of a Graafian follicle along with androgen-secreting theca interna cells and estrogen-secreting granulosa cells.

Interestingly, reports revealed that the serum leptin levels were suppressed in ovarian tumour-associated hyperandrogenism (i.e., excess levels of androgens like testosterone) (Pekic et al., 2001; Cvijovic et al., 2007). It is worth mentioning that ovarian sex cord-stromal tumours such as Sertoli-Leydig cell and steroid cell tumours cause hyperandrogenism (Tanaka et al., 2004). Nevertheless, the effects of androgens on leptin are highly intricate (Wildman et al., 2013; Nohara et al., 2014; Iwasa et al., 2016). Iwasa and colleagues continuously administered testosterone in Sprague-Dawley female adult rats by implanting a silastic tube filled with crystalline testosterone (Iwasa et al., 2016). In their study, visceral fat leptin mRNA levels were significantly lower in the testosterone-administered group than in the control group. On the other hand, different clinical reports have recorded that ovarian surface epithelial neoplasms can coexist with sex cord-stromal tumours or both categories of neoplastic lesions may show similarities (Seo et al., 1996; Stacher et al., 2010; Singh et al., 2014; Young, 2018). It is known that the stromal component cells such as the theca interna cells biosynthesize androgens under the influence of luteinizing hormone (LH) and the granulosa cells produce oestrogens from androgens in response to follicle-stimulating hormone (FSH) (Figure 3). Therefore, in pathological situation, the involvement of androgen-secreting cells or excess release of androgens may lead to the suppression of leptin.

In line with the abovementioned trend, Visintin et al. (2008) documented low serum levels of leptin among patients with ovarian cancer compared with healthy women. Overall, they analyzed 156 women with newly diagnosed ovarian cancer (stage I/II disease: 36, III/IV: 120) and 362 age-matched healthy controls. In a study conducted by Macciò et al. (2009), the serum levels of leptin were significantly lower in patients with stage III/IV ovarian cancer (n=75) in comparison with controls (n= 95) and patients with stage I/II disease (n=29). Likewise, in another study, the plasma level of leptin was decreased in patients with ovarian cancer (n=151) compared to healthy controls (n=75) (Lu et al., 2011). Apart from the similar findings like lower serum leptin concentrations in ovarian cancer patients than controls (n=51), the investigators of a study also found that patients with stage III/IV (n=38) had lower mean serum leptin levels in comparison to patients with I/II stage (n=15) (Grabowski et al., 2014). Furthermore, they observed significant elevation of mean post-operative serum leptin concentrations in patients who underwent complete tumour resection.

A number of studies did not detect any statistically significant changes in serum leptin levels between patients and controls (Serin et al., 2008; Mrochem et al., 2008; Hwang et al., 2009; Vysotskii et al., 2009). Although patients’ leptin levels were similar to healthy women in the study of Vysotskii et al. (2009), leptin levels in patients with poorly and moderately differentiated serous ovarian cancer were 2-fold higher than in well-differentiated tumours. In addition, serum concentrations of leptin increased with the progression of disease stage (Vysotskii et al., 2009). On the other hand, Wu et al. observed that patients with ovarian cancer had a significantly higher level of leptin in the plasma samples compared to their matched controls in the Community-Based Cancer Screening Program (CBCSP, Taiwan) study (Wu et al., 2014). In this study, 30 cases of ovarian cancer were diagnosed in the cohort of 11,258 women during a median follow-up of 19.9 years.

In a study conducted by Kato et al. (2015), Ob-Rb protein expression levels were evaluated in primary tumours, cases with ascites, and metastatic tumours. A worse overall survival rate was found in patients expressing higher leptin/Ob-Rb levels. Ob-Rb was highly expressed in cases with ascites and metastases than in primary tumours. Moreover, serum and ascitic fluid leptin levels were higher in overweight patients experiencing worse survival. After analyzing the ascitic fluid samples from a total of 48 patients with epithelial ovarian cancer, Matte et al. (2012) detected elevated levels of leptin, which were associated with shorter progression-free survival. Using a different approach, Diaz et al. (2013) analyzed pre-diagnostic serum samples of a cohort of 161 women with advanced stage epithelial ovarian cancer. In their study, women with low leptin to adiponectin (L:A) ratios demonstrated longer disease-specific survival compared to those with median or high levels (Diaz et al., 2013).

Several investigators have evaluated the immunoexpression of Ob-R and/or leptin in tumour tissue samples and tried to ascertain any relationships with the disease processes (Uddin et al., 2009; Kato et al., 2015; Kumar et al., 2017). After analyzing 156 patients with epithelial ovarian carcinoma, Uddin et al. (2009) noticed the overexpression of Ob-R in 59.2% of cases; this overexpression was significantly associated with poor progression-free survival. Furthermore, in their study, Ob-R expression was associated with the expression of the anti-apoptotic proteins, Bcl-XL and XIAP. Similarly, in an Australian study, a statistically significant decrease in survival was identified for patients with high expression of both leptin and Ob-R (Kumar et al., 2017).

In normal ovaries, leptin probably has a diverse range of functions. Generally ovarian cancers arise in the surface epithelial cells, and the majority of studies pertaining to leptin have focused on this group of malignancies. Different ovarian cancer cell lines usually have shown cellular growth and proliferation in response to leptin treatment. However, unlike other cancers, a lower mean level of circulating leptin has been observed commonly among patients with epithelial ovarian cancer in comparison to non-cancerous or healthy controls. Conversely, studies have suggested that high expression of leptin and its receptors in tumours possibly indicate poor prognosis.

## Conclusions

Being a hormone producing organ, the ovary has a huge influence over the endometrium in different physiological and pathological conditions. In addition, there is a close relationship/similarity between these two organs in several pathologies, e.g., increased risk of endometrial cancer in PCOS, histological similarities in certain endometrial and ovarian cancers like endometrioid morphology, synchronous endometrial and ovarian cancers including metastatic spread in both directions from primary tumours. It is worthy of note that oestrogen is an important contributing agent for the development of endometrial cancer. In both pre- and post-menopausal situations, the ovaries play a key role in the biosynthesis of oestrogens. In the postmenopausal state, hyperplasia of ovarian stroma is associated with an increased androgen production and oestrogens originate from the peripheral conversion of androgens, which lead to the development of endometrial pathology (Jongen et al., 2002). Studies conducted from various approaches identified a complex relationship between oestrogen and leptin (Laughlin et al., 2006; Karamouti et al., 2008; Choi et al., 2011; Jones et al., 2013; Liu et al., 2013; Qian et al., 2015; Hoffmann et al., 2016). Interestingly, a study noticed high levels of Ob-R protein expression in endometrial biopsies collected from ovarian cancer patients (Méndez-López et al., 2013). The abovementioned phenomena indicate a highly intricate nature of leptin signalling and its complex interactions with other hormones, such as oestrogens. An appropriate knowledge of leptin’s intracellular crosstalk could be helpful in understanding the disease risk and early detection, particularly for the neoplastic growth of the ovary, which is usually diagnosed at a late stage and prognosis remains poor.
